# 4‐O‐methylhonokiol protects against diabetic cardiomyopathy in type 2 diabetic mice by activation of AMPK‐mediated cardiac lipid metabolism improvement

**DOI:** 10.1111/jcmm.14493

**Published:** 2019-06-14

**Authors:** Zongyu Zheng, Tianjiao Ma, Hua Guo, Ki Soo Kim, Kyoung Tae Kim, Liqi Bi, Zhiguo Zhang, Lu Cai

**Affiliations:** ^1^ Departments of Urology and Cardiology The First Hospital of Jilin University Changchun China; ^2^ Department of Pediatrics Pediatric Research Institute, University of Louisville Louisville Kentucky; ^3^ Department of Rheumatology and Immunology China‐Japan Union Hospital of Jilin University Changchun China; ^4^ Department of Immunology, Zhejiang Key Laboratory of Pathophysiology Medical School of Ningbo University Ningbo China; ^5^ SK Bioland Haimen Co. LTD Haimen China; ^6^ Department of Radiation Oncology, Pharmacology and Toxicology University of Louisville Louisville Kentucky

**Keywords:** 4‐O‐methylhonokiol, AMPK, diabetic cardiomyopathy

## Abstract

Diabetic cardiomyopathy (DCM) is characterized by increased left ventricular mass and wall thickness, decreased systolic function, reduced ejection fraction (EF) and ultimately heart failure. The *4‐O‐methylhonokiol* (MH) has been isolated mainly from the bark of the root and stem of Magnolia species. In this study, we aimed to elucidate whether MH can effectively prevent DCM in type 2 diabetic (T2D) mice and, if so, whether the protective response of MH is associated with its activation of AMPK‐mediated inhibition of lipid accumulation and inflammation. A total number of 40 mice were divided into four groups: Ctrl, Ctrl + MH, T2D, T2D + MH. Five mice from each group were sacrificed after 3‐month MH treatment. The remaining animals in each group were kept for additional 3 months without further MH treatment. In T2D mice, the typical DCM symptoms were induced as expected, reflected by decreased ejection fraction and lipotoxic effects inducing lipid accumulation, oxidative stress, inflammatory reactions, and final fibrosis. However, these typical DCM changes were significantly prevented by the MH treatment immediately or 3 months after the 3‐month MH treatment, suggesting MH‐induced cardiac protection from T2D had a memory effect. Mechanistically, MH cardiac protection from DCM may be associated with its lipid metabolism improvement by the activation of AMPK/CPT1‐mediated fatty acid oxidation. In addition, the MH treatment of DCM mice significantly improved their insulin resistance levels by activation of GSK‐3β. These results indicate that the treatment of T2D with MH effectively prevents DCM probably *via* AMPK‐dependent improvement of the lipid metabolism.

## 1 INTRODUCTION

Obesity is becoming a serious health issue all over the world.^1^ In 2016, 39% of the adults of the global population (1.9 billion people aged 18 years and older) were overweighed (body‐mass index ≥25 kg/m^2^). Of them, over 13% (650 million adults) were obese (body‐mass index ≥30 kg/m^2^).^2^ As the number of overweighed and obese people increases, a dramatically rising trend is being observed in the number of patients with type 2 diabetes (T2D). Recent estimates showed a number of almost 422 million of adults living with diabetes in 2014.^3^

Diabetic cardiomyopathy (DCM) is characterized by an increased left ventricular mass and wall thickness,^4^ diastolic dysfunction, decreased systolic function, reduced ejection fraction (EF), and ultimately heart failure.^5^ Several mechanisms that may be responsible for DCM have been proposed in an earlier investigation.^6^ For instance, intracellular calcium handling may be impaired, resulting in a loss of cardiac contractility impaired. Moreover, the excessive production of reactive oxygen and nitrogen species (ROS/RNS) leads to tissue damage and cell death; extra cellular fatty acids (FA) metabolism and excess energy generation can result in lipotoxicity in the heart. Additionally, the accumulation of advanced glycation end‐products in the heart causes accumulation of extracellular matrix (ECM) and cardiac fibrosis, which can consequentially cause cardiac dysfunction and eventually heart failure.

In diabetes, cardiac intracellular FA oxidation was found to increase, whereas glucose oxidation was inhibited.^6^ Evidence exists that the cardiac function in DCM can be ameliorated by reducing FA oxidation or increasing the glucose oxidation to regulate the cardiac energy metabolism.^7^ There are two scenarios that can explain intramyocardial lipid accumulation: impaired FA oxidation and FA oversupply.^8^ Carnitine palmitoyltransferase 1 (CPT1) level, a key enzyme regulating the entry of FA into the mitochondria for oxidation, was decreased in T2D cardiomyopathy, which may contribute to mitochondrial FA oxidation impairment. The FA transporters CD36 and FA oxidation enzymes peroxisome proliferator activated receptor‐α (PPARα) are upregulated in diabetic hearts which could contribute to the FA oversupply in cardiac, resulting in accumulation of intramyocardial lipids.^9^

Previous reports indicate that *Magnolia* have a wide range of biological activities such as anti‐inflammatory and antioxidative.^10^ The bioactive substance 4‐O‐methylhonokiol (MH) was isolated mainly from the bark of the root and stem of *Magnolia* species.^11^ In our previous study, we found that MH reduced plasma TG and cholesterol levels and significantly improved high‐fat diet (HFD)‐induced insulin resistance in obese mice. In earlier investigations MH suppressed lipid accumulation and adipose tissue inflammation in C57BL/6J mice.^12^ Furthermore, we discovered that MH prevented obesity‐induced heart damage by inhibiting CD36 expression and activating the AMPK‐Sirt1‐PGC‐1α pathway in HFD‐induced obese mice by 13

In our previous study,^13^ we discovered that MH avoided obesity‐induced mild heart damage in obese mice at the time after 3‐month MH treatment. However, the morbidity of diabetes, particularly T2D has significantly increased and DCM is one of the major complications of T2D. In this study, therefore, we aimed to clarify whether MH can effectively prevent DCM in T2D and also whether MH‐mediated cardiac protection from diabetes remains persistent at certain times after MH treatment. Moreover, if a preventive effect was established, our purpose was to find whether this prophylactic effect of MH was associated with AMPK‐mediated abnormal lipid metabolism and inflammation‐inhibiting activation.

## 2 MATERIALS AND METHODS

### 2.1 Experimental protocols and animals

Seven‐week‐old C57BL/6J male mice were purchased from the Jackson Laboratory, and experiments were conducted at the University of Louisville Research Resources Center (Louisville, KY, USA). The experimental mice were maintained at a temperature of 22°C, under a 12‐hour light/dark cycle, and ad libitum access to food and water. All animal experiments were approved by the Institutional Animal Care and Use Committee of the University of Louisville, accredited by the American Association for Accreditation of Laboratory Animal Care. All experiments involving mice were in compliance with the guidelines of the United States National Institutes of Health for the Care and Use of Laboratory Animals (NIH publication, revised 2011).

T2D is characterized by significant insulin resistance and lack of insulin secretion.^14^ In our study, the mice were HFD‐fed for 3 months to produce insulin resistance,^15^ followed by the application of a single dose of streptozotocin (STZ) (100 mg/kg) (Sigma‐Aldrich, St. Louis, MO, USA), which achieved a model with insulin resistance, mild lack of insulin secretion and hyperglycemia, a T2D mouse model, as previously described.^16^ The mice were fed normal diet (ND, 16.8 kcal% fat; 3.3 kcal/g, TD.120455, Teklad Custom Research Diets, Envigo, Huntingdon, UK) or HFD (60.3 kcal% fat; 5.1 kcal/g, TD.09766, Teklad Custom Research Diets, Envigo). Diabetic mice (3‐h fasting blood glucose level ≥220 mg/dL) were defined after one‐week STZ administration. Then, the diabetic and age‐matched controls (non‐STZ treated) mice were divided into four groups: Ctrl, Ctrl +MH, T2D, and T2D + MH. Mice were gavage‐fed 1.0 mg/kg MH or vehicle for five consecutive days a week for 3 months while continuously feeding HFD or ND, based on previous studies from our own 13 and others.^17^ Upon completion of the 3‐month MH treatment, five mice in each group were sacrificed, and their heart tissues were collected and marked as obtained at the 3‐month time point. The animals remaining in each group were maintained for another 3 months of HFD or ND feeding without further MH treatment. Finally, all mice were sacrificed, and their cardiac tissues were collected and labeljed as taken at the 6‐month time point. The inclusion of additional observation of MH‐treated or non‐treated T2D mice for 3 months without MH treatment is to determine whether MH‐mediated cardiac protection from T2D is long‐lasting persist or not.

### 2.2 Echocardiography

Transthoracic echocardiography was performed using a Vevo 770 ultrasound system (VisualSonics Inc, Toronto, ON, Canada) by applying a high‐frequency ultrasound probe (RMV‐707B). The echocardiography procedure is described in detail in our previous reports.^13^ The left ventricular posterior wall thickness end diastole (LVPW; d) and end systole (LVPW; S), left ventricular volume end diastolic (LV vol; d) and end systolic (LV vol; s:), left ventricular internal diameter end diastole (LVID; d) and end systole (LVID; s), ejection fraction (EF), fractional shortening (FS), and LV mass were measured using the LV M‐Mode images. The data were averaged for 10 cardiac cycles.

### 2.3 Oil Red O staining for lipid accumulation

Oil red O staining was performed to check lipid accumulation in the heart. Briefly, after cryostat sectioning, heart tissue sections were fixed in formalin, rinse with running tap water, immersed in isopropanol and then stained with Oil Red O (saturated oil red O isopropanol solution diluted 4:6 to 60% isopropanol, Sigma‐Aldrich, St. Louis, MO).^14^

### 2.4 Sirius red staining

A previously established histopathological protocol was used.^18^ Sirius Red staining was applied for collagen and fibronectin deposition determination.^19^ The stained sections were then viewed using a Nikon Eclipse E600 microscope (Tokyo, Japan).

### 2.5 Quantitative analysis of lipid peroxides

The lipid peroxide concentration was determined as previously described by our group. This method examines thiobarbituric acid reactivity by measuring the amount of malondialdehyde (MDA) formed during acid hydrolysis of the lipid peroxide compound.^16^

### 2.6 Western blot analysis

Western blotting was performed as previously described.^19^, ^20^ The following primary antibodies were used: β‐Actin, ICAM, IL‐1β, Sirt1 (Santa Cruz Biotechnology, Dallas, TX, USA); PGC‐1α, CPT‐1B, phospho‐GSK‐3β (p‐GSK‐3β), total‐GSK‐3β (t‐GSK‐3β), phospho‐AMPKα (p‐AMPKα), total‐AMPKα (t‐AMPKα) (Cell Signaling Technology, Beverly, MA, USA); 3‐nitrotyrosine (3‐NT, Millipore Corp., Temecula, CA); Anti‐4 hydroxynonenal (4‐HNE, Alpha Diagnostic Int.) and PPARα, CD36, Laminin, FN, TNF‐α, SOD2 and heme oxygenase‐1 (HO‐1) (Abcam, Cambridge, MA, USA). The corresponding secondary antibody and β‐Actin were employed as an internal control.

### 2.7 Statistical analysis

Data were collected from five animals per group and data are presented as the mean ± SD. Statistical analysis was calculated using One‐way Analysis of Variation (ANOVA) with the Tukey's test by using the GraphPad Prism 6 software (GraphPad Software Inc, San Diego, CA, USA). Values of *P* < 0.05 were considered statistically significant.

## 3 RESULTS

### 3.1 MH treatment prevents diabetes‐induced cardiac dysfunction in T2D mice

**Figure 1:**
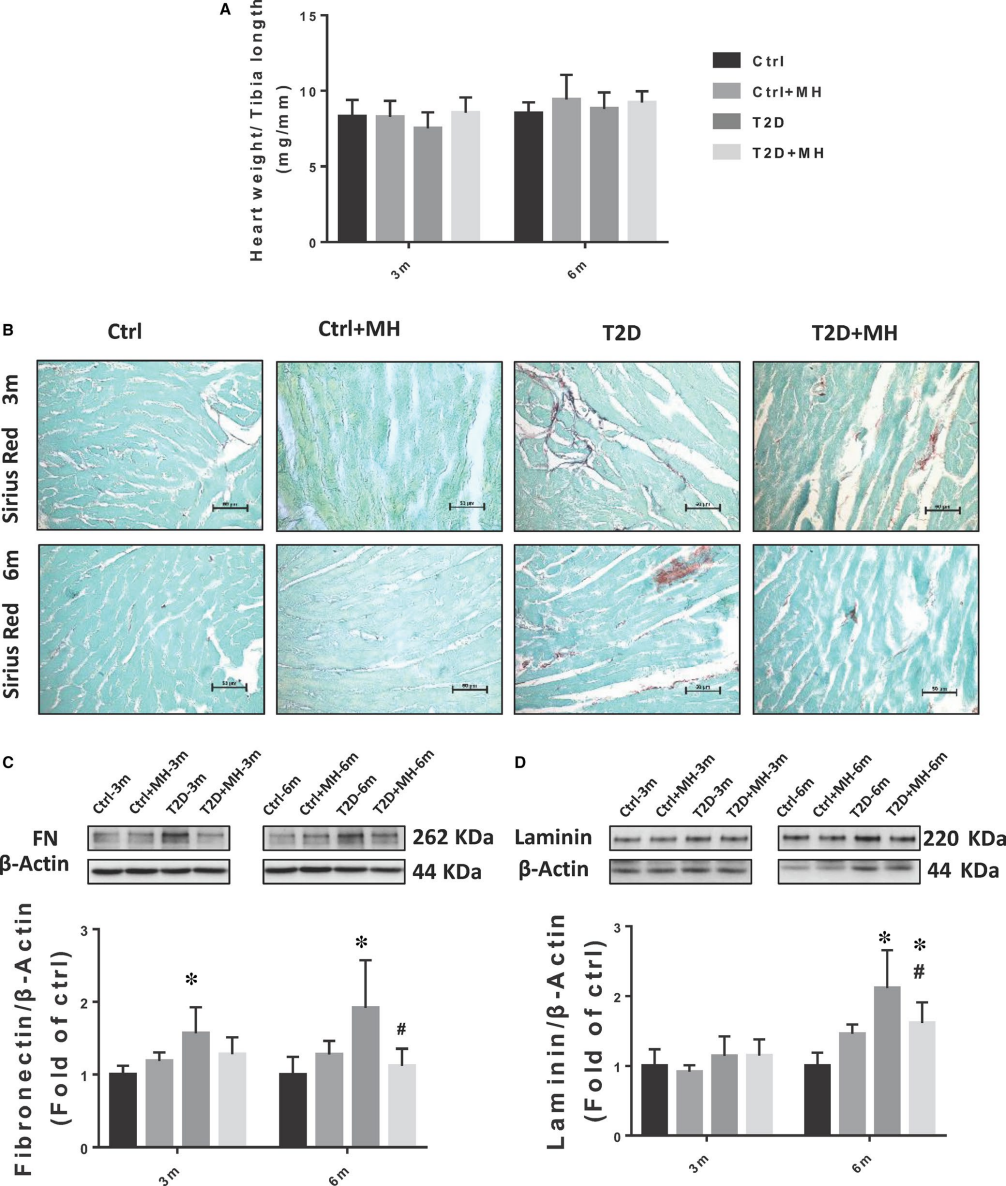
MH treatment for prevention of diabetes‐induced cardiac fibrosis. (A) Ratio of heart weight to body weight(mg/mm). (B) Representative images of Sirius Red staining. (C, D) Representative results and densitometric analyses of Western blot for FN and Laminin protein in the heart. Data are represented as mean ± SD (n = 5 in each group). Statistical significance is considered at *P < 0.05, versus Ctrl, #*P* < 0.05, versus T2D at the same time point

Blood glucose (BG) levels were significantly elevated in all T2D mice compared to that in the ND‐fed Ctrl mice (Table 1). MH significantly ameliorated high BG induced by DM in 6 months. All T2D mice did not exhibit significant change for the heart weight‐to‐tibia length ratio compared to Ctrl mice (Figure 1A).

**Table 1:** Fasting blood glucose (mg/dL)

	Ctrl	Ctrl + MH	DM	DM + MH
Model setup	116.4 ± 31.2	129.4 ± 12.2	257.0 ± 23.6^*^	275.2 ± 66.7^*^
2 months	104.4 ± 9.4	111.4 ± 11.1	148.4 ± 30.1	172.2 ± 39.2^*^
4 months	105.0 ± 14.6	121.2 ± 31.9	159.6 ± 34.8^*^	227.0 ± 40.5^*^
6 months	130.2 ± 24.4	142.2 ± 38.6	196.8 ± 29.5^*^	141.6 ± 23.3

Data are represented as mean ± SD (n = 5 in each group). Statistical significance is considered at *P < 0.05 vs Ctrl #*P* < 0.05 versus T2D at the same time point.

Echocardiographic measurements (Tables 2 and 3) showed a significant increase in the left ventricular posterior wall end‐diastolic wall thickness (LVPW; d) at the 6‐month time point in T2D mice, suggesting left ventricular hypertrophy. The percentages of ejection fraction (EF) and fractional shortening (FS) in the T2D mice were decreased, whereas the left ventricular end‐systolic volume (LV vol; s) was increased at the 3‐ and 6‐month time points, implying a decrease in the left ventricular systolic function in the T2D mice. At the 3‐month time point, the MH treatment significantly reduced the elevation in left ventricular end‐systolic volume in the T2D mice, inhibiting the decline in EF and FS. At the 6‐month time point, the MH treatment significantly suppressed the decline in EF and FS and the augmentation in the left ventricular posterior wall thickness end‐diastolic diameter. In addition, there was a slight increase in LV mass in T2D mice compared to Ctrl mice, but there was no statistical difference.

**Table 2:** Cardiac dysfunction induced by T2D was attenuated by the MH treatment in 3 months

	Ctrl‐3m	Ctrl + MH‐3m	DM‐3m	DM + MH‐3m
LVPW;d(mm)	0.68 ± 0.02	0.70 ± 0.04	0.72 ± 0.02	0.72 ± 0.01
LVPW;s(mm)	1.10 ± 0.07	1.05 ± 0.05	1.03 ± 0.11	1.05 ± 0.03
LVID;d (mm)	4.20 ± 0.21	4.01 ± 0.19	4.25 ± 0.18	4.07 ± 0.18
LVID;s (mm)	2.72 ± 0.18	2.57 ± 0.15	2.93 ± 0.12	2.67 ± 0.12
LV vol;d (mm)	78.87 ± 9.17	70.84 ± 8.04	81.23 ± 7.99	73.31 ± 7.21
LV vol;s (mm)	27.06 ± 3.60	23.92 ± 3.27	33.45 ± 2.58^*^	26.34 ± 3.04^#^
%EF	65.07 ± 2.30	66.30 ± 1.61	59.33 ± 1.90^*^	64.10 ± 1.22^#^
%FS	35.32 ± 1.65	36.12 ± 1.18	31.22 ± 1.90*	34.50 ± 0.89^#^
LV mass (mg)	76.68 ± 7.91	72.01 ± 8.74	82.01 ± 7.93	75.98 ± 5.08

Abbreviations: EF: ejection fraction; FS: fractional shortening; LVID; d: left ventricular internal diameter end diastole; LVID; s: left ventricular internal diameter end systole; LV mass: left ventricular mass; LVPW: left ventricular posterior wall; LV vol; d: left ventricular end diastolic volume; LV vol; s: left ventricular end systolic volume. Data are represented as mean ± SD (n = 5 in each group).

Statistical significance is considered at *P < 0.05, versus Ctrl #*P* < 0.05, vs T2D at the same time point.

**Table 3:** Cardiac dysfunction induced by T2D was attenuated by the MH treatment in 6 months

	Ctrl‐6m	Ctrl + MH‐6m	DM‐6m	DM + MH‐6m
LVPW;d(mm)	0.69 ± 0.01	0.69 ± 0.01	0.76 ± 0.02^*^	0.72 ± 0.02^#^
LVPW;s(mm)	1.10 ± 0.02	1.11 ± 0.04	1.02 ± 0.05^*^	1.07 ± 0.04
LVID;d (mm)	4.24 ± 0.10	4.29 ± 0.25	4.33 ± 0.07	4.20 ± 0.18
LVID;s (mm)	2.70 ± 0.09	2.77 ± 0.19	2.93 ± 0.08	2.75 ± 0.14
LV vol;d (mm)	80.35 ± 4.51	83.15 ± 11.52	84.28 ± 3.48	78.79 ± 7.90
LV vol;s (mm)	27.11 ± 2.33	27.69 ± 2.76	33.11 ± 2.09^*^	28.40 ± 3.37
%EF	66.26 ± 2.26	65.18 ± 1.76	60.74 ± 1.27^*^	64.13 ± 1.16^#^
%FS	36.26 ± 1.73	35.45 ± 1.22	32.25 ± 0.87^*^	34.72 ± 0.76^#^
LV mass (mg)	80.03 ± 4.95	81.61 ± 9.66	87.69 ± 2.36	80.41 ± 4.58

Abbreviations: EF: ejection fraction; FS: fractional shortening; LV mass: left ventricular mass; LV vol; d: left ventricular end diastolic volume; LV vol; s: left ventricular end systolic volume; LVID; d: left ventricular internal diameter end diastole; LVID; s: left ventricular internal diameter end systole; LVPW: left ventricular posterior wall. Data are represented as mean ± SD (n = 5 in each group).

Statistical significance is considered at *P < 0.05, versus Ctrl #*P* < 0.05, versus T2D at the same time point.

### 3.2 MH treatment prevents diabetes‐induced cardiac fibrosis in T2D mice

Fibrosis has been suggested to play an important role in diabetes‐induced DCM.^21^ In this investigation, we aimed to clarify whether the administration of MH prevents cardiac fibrosis in T2D mice. Using Sirius Red staining, we observed a significant increase in the collagen deposition in T2D mice as indicated by the red‐stained areas in the myocardial stroma at both 3 and 6 months. However, collagen deposition was significantly attenuated by MH (Figure 1B). Similarly, consistent with the results of the Sirius Red staining, the cardiac fibrosis marker FN, measured by Western blot, was significantly increased in T2D mice at 3 and 6 months (Figure 1C). In addition, the level of the fibrotic biomarker Laminin was increased in T2D mice at 6 months (Figure 1D), which was significantly reduced by MH treatment.

### 3.3 MH treatment prevents diabetes‐induced cardiac inflammation and oxidative stress in T2D mice

**Figure 2:**
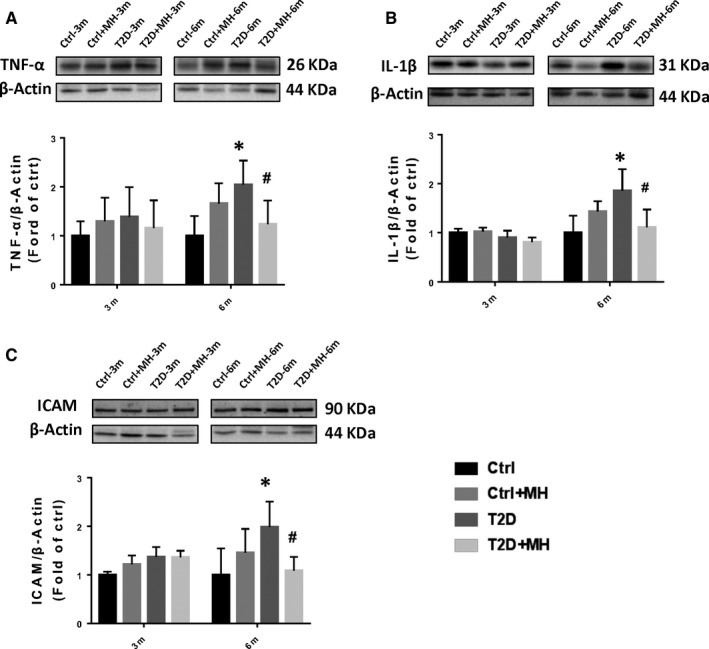
MH treatment for prevention of diabetes‐induced cardiac inflammation. (A–C) Representative results and densitometric analyses of Western blot for, TNF‐α (A), IL‐1β (B), and ICAM (C) protein in the heart. Data are represented as mean ± SD (n = 5 in each group). Statistical significance is considered at *P < 0.05, versus Ctrl, ＃*P* < 0.05, versus T2D at the same time point.

Inflammation and oxidative stress were the main pathological consequences of diabetes‐induced DCM. Increasing evidence suggests that inflammation and oxidative stress promote each other in T2D. Western blot results showed that the expression of other classical inflammatory markers TNF‐α (Figure 2A), IL‐1β (Figure 2B), and ICAM (Figure 2C) in the cardiac tissue were not significantly changed at the 3‐month time point. However, their expression was significantly increased in T2D mice at the 6‐month time point, whereas the MH treatment prevented cardiac inflammation in T2D mice.

**Figure 3:**
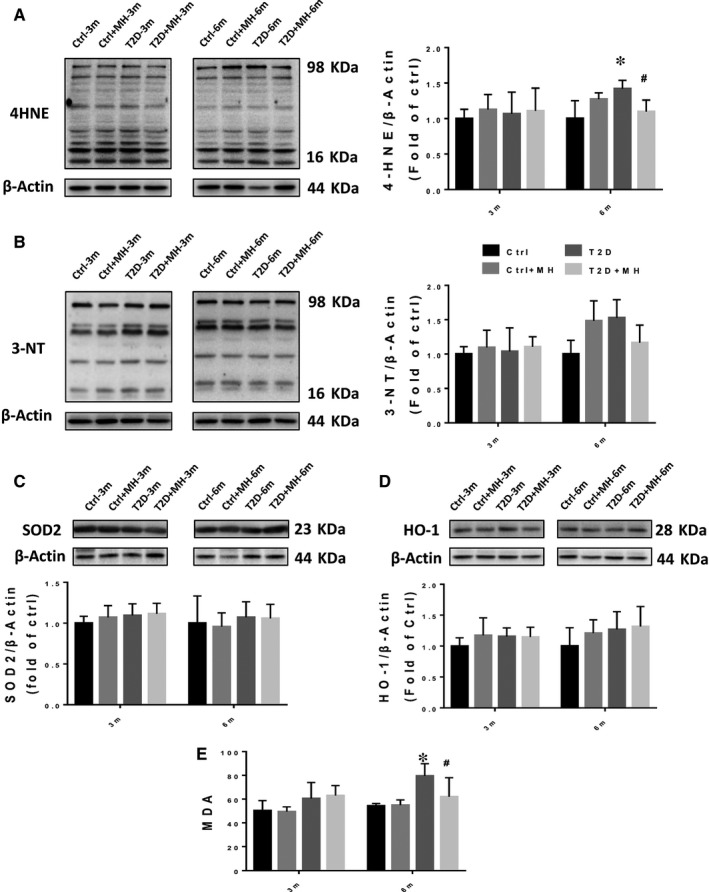
MH treatment for prevention of diabetes‐induced cardiac oxidative stress. (AD) Representative results and densitometric analyses of Western blot for 4‐HNE (A) 3‐NT (B), SOD2 (C) and HO‐1 (D) protein in the heart. Lipid peroxidation with MDA assay (E). Data are represented as mean ± SD (n = 5 in each group). Statistical significance is considered as *P < 0.05, versus Ctrl, #*P* < 0.05, versus T2D at the same time point

T2D significantly increased the cardiac oxidative stress, assessed by quantifying the lipid peroxidation indicator 4‐HNE (Figure 3A) at 6 months, which was completely prevented by the MH treatment. No significant difference in the nitrosative stress indicator, 3‐NT expression, was established between the 3‐ and 6‐month time points (Figure 3B). Western blot data showed that the expression of SOD2 and HO‐1 had no significant change at 3‐month and 6‐month time point (Figure 3C,3). Lipid peroxidation was detected by MDA assay (Figure 3E). Oxidative stress indices were significantly increased in the T2D mice which was decreased by MH treatment at 6‐month time point.

### 3.4 Preventive effect of MH on the regulation of lipid metabolism dysfunction on T2D mice

**Figure 4:**
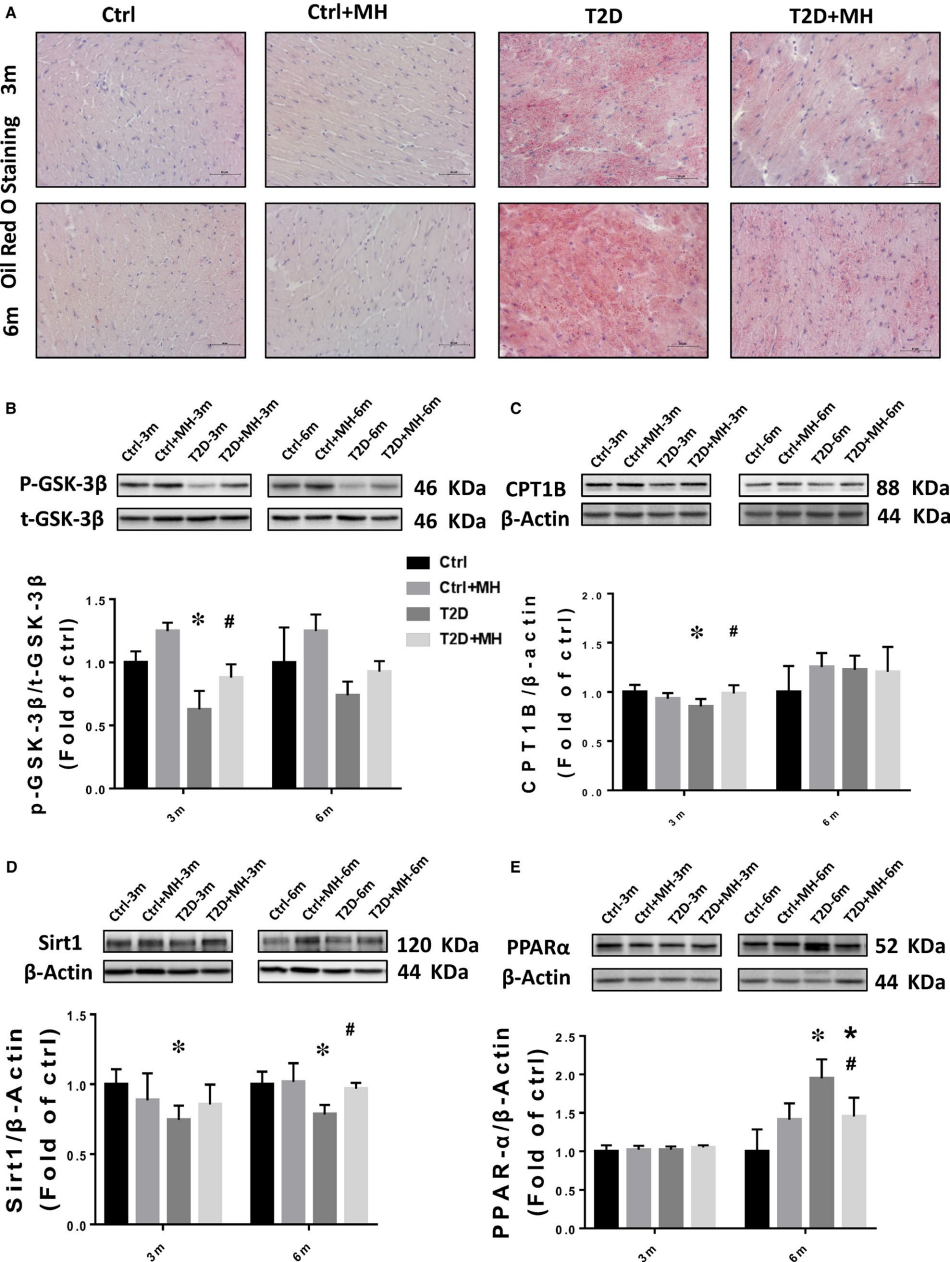
MH attenuated diabetes‐induced lipid dysfunction in the cardiac tissue. (A) Heart lipid accumulation was detected by Oil Red O staining (original magnification, 40×) in cryosections. (B–E) Representative results and densitometric analyses of Western blot for GSK‐3β (B), CPT1B (C), Sirt1 (D), and PPARα (E) protein in the heart. Data are represented as mean ± SD (n = 5 in each group). Statistical significance is considered at *P < 0.05, versus Ctrl, #*P* < 0.05, versus T2D at the same time point

**Figure 5:**
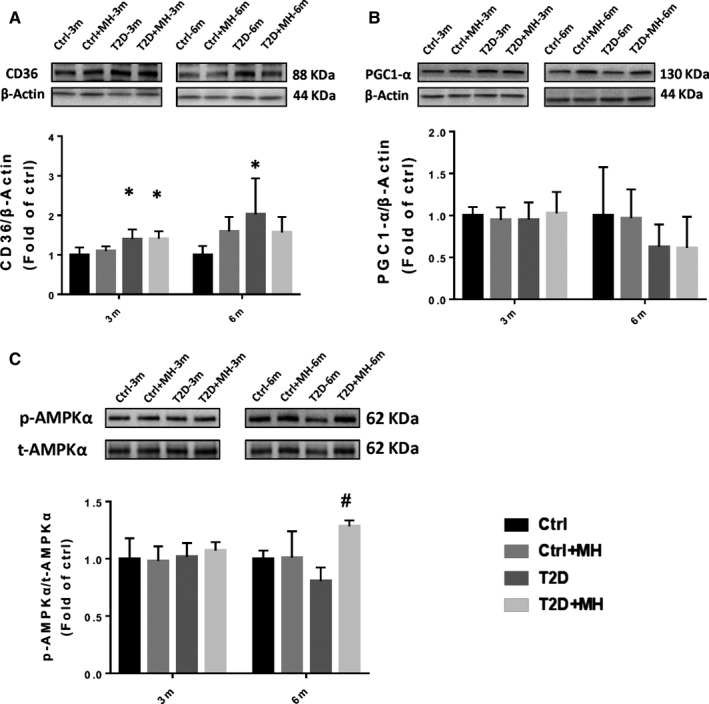
MH attenuated diabetes‐induced changes of CD36, PGC1‐α, and p‐AMPKα in the cardiac tissue. (A–C) Representative results and densitometric analyses of Western blot for CD36 (A), PGC1‐α (B), and p‐AMPKα (C) protein in the heart. Data are represented as mean ± SD (n = 5 in each group). Statistical significance is considered at *P < 0.05, versus Ctrl, #*P* < 0.05, versus T2D at the same time point

T2D is associated with obesity and increased myocardial lipid β‐oxidation. We used the Oil Red O staining to measure lipid accumulation in the heart. It showed a significant increase in lipid accumulation in the myocardial muscle of T2D group, compared to Ctrl group. MH treatment markedly decreased lipid accumulation in the heart (Figure 4A). To understand how MH regulates the lipid metabolism in the heart, we examined GSK‐3β, which has been previously associated with obese animal models and obese human T2D skeletal muscle insulin resistance.^22^ Our results showed that the phosphorylation of GSK‐3β was decreased in the T2D‐induced diabetic cardiomyocytes, whereas the MH treatment restored the level of p‐GSK‐3β in T2D‐induced diabetic cardiomyocytes at the 3‐month time point (Figure 4B). We assessed the expression of a mitochondrial enzyme, carnitine palmitoyltransferase I (CPT1). More specifically, we used CPT1B, one of the predominant CPT1 isoforms that are predominantly expressed in the heart.^23^ We found that T2D significantly decreased the expression of CPT1B. The treatment of T2D mice with MH significantly reduced the effect of T2D on CPT1B expression at the 3‐month time point (Figure 4C). Consistent with our previous studies, the Sirt1 expression was significantly decreased in T2D at 3 and 6 months.^24^ Nevertheless, the MH treatment restored the level of Sirt1 in T2D‐induced diabetic cardiomyocytes at 6 months (Figure 4D). Next, we assessed the expression of transcription factor (PPARα), FA uptake protein (CD36), and controlling the lipolysis protein PGC‐1α. The results of the Western blot assay revealed that T2D significantly up‐regulated the expression of PPARα at 6 months (Figure 4E). The MH treatment significantly attenuated the T2D‐induced upregulation of PPARα, whereas no significant differences among the experimental groups at 3 months were observed. At the 3‐month time point, CD36 increased more significantly in the T2D group than in the Ctrl group. We found that the MH treatment had a tendency to lower CD36 expression at the 6‐month time point, without statistical significance (Figure 5A). At the 3‐ and 6‐month time points, no significant difference was detected in the expression of PGC1‐α between the groups (Figure 5B). The MH treatment activated AMPKα in T2D mice at the 6‐month time point (Figure 5C).

## 4 DISCUSSION

**Figure 6:**
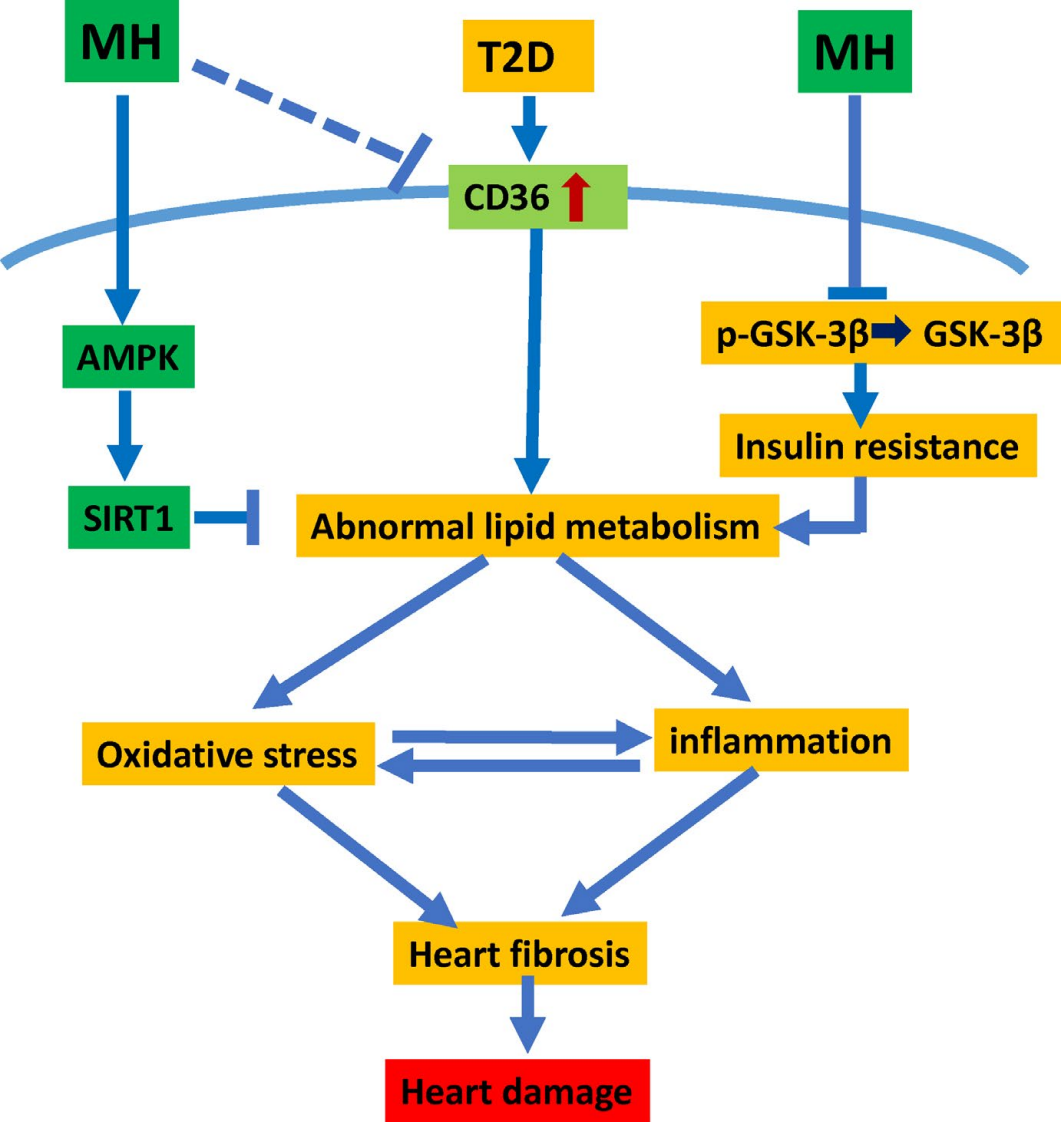
The mechanism of action of MH on diabetic cardiomyopathy. On the one hand, MH regulates intracellular lipid accumulation and regulates the lipid metabolism and modulates PPARα by activating the AMPK‐Sirt1 pathway. On the other hand, MH regulates insulin resistance by GSK‐3β. Finally, MH protects the heart of T2D by modulating lipid metabolism, mitigating oxidative stress and inflammation, and acting as an antioxidant, anti‐inflammatory and anti‐fibrotic agent

To date, increasing evidence has suggested that abnormal lipid metabolism and inflammation are critically involved in the development of DCM. Our results show the following important findings: (a) this is the first in vivo study on the role of MH in the prevention of T2D‐induced cardiac injury; (b) the administration of MH in T2D mice effectively attenuated cardiac lipid dysfunction, fibrosis, oxidative stress and inflammation. As illustrated in Figure 6, the main mechanism leading to these favorable results may be related to MH activation of the AMPK function, which in turn activates AMPK/Sirt1 pathways and ameliorates the T2D‐induced lipid metabolism abnormalities; (c) the protective effect against DCM remained 3 months after MH withdrawal. All these results indicate the protective potential of MH against DCM. In our previously published study, we reported that MH prevented HFD‐induced cardiac pathogenesis by reduced cardiac lipid accumulation and decreased cardiac FA transcriptase/CD36 protein expression.^13^ Here, we expanded our investigation to a T2D model, focusing on elucidating the main mechanism by which MH attenuated abnormal lipid metabolism and inflammation.

DM can cause cardiac remodeling and structural changes by activating the renin‐angiotensin system, leading to LV dysfunction, including diastolic dysfunction and systolic dysfunction.^25^, ^26^ The results of our HFD + STZ‐induced cardiac dysfunction in T2D mice are consistent with the above evidence. We established left ventricular hypertrophy and decreased left ventricular systolic function in the T2D mice, which indicates cardiac dysfunction. At 3 months, these changes in the cardiac morphology and function in T2D mice were attenuated by MH to varying degrees. Importantly, 3 months after MH withdrawal, the protection against DCM was retained.

Cardiac fibrosis is a hallmark of structural cardiac remodeling in diabetes and is primarily due to the gradual accumulation of ECM components. The T2D group mice exhibited higher fibrosis levels than the Ctrl group as revealed by the Sirius Red staining. The MH treatment reduced the red area of fibrosis. ECM has been found to be composed mainly of collagen, fibronectin, and laminin.^27^ Therefore, we evaluated the expression of FN and laminin in the heart. A significant increase in the expression of FN and laminin in the hearts of the T2D group was detected, which was decreased by MH treatment. MH was previously reported to be able to reduce fibrosis via suppressing the CTGF and TGF‐ β1 expression in HFD‐reduced liver fibrosis.^28^ This study results reveal that MH exerts significant anti‐fibrotic effects in DCM in T2D mice.

Oxidative stress can promote heart inflammation, while, in turn, inflammation can also enhance the further increase in oxidative stress.^29^ Chronic inflammatory response in diabetic‐induced cardiac tissue leads to pro‐inflammatory cytokines (TNF‐α, IL‐1β) and cell adhesion molecules (ICAM) increased. Previous research showed that *Magnolia kobus* potently inhibited the lipopolysaccharide‐induced production of TNF‐α and IL‐1β in a murine macrophage‐like cell.^30^ Our current results indicate that T2D induced significant increases in TNF‐α, IL‐1β, and ICAM expression levels, which were substantially reduced by MH.

Oxidative stress was closely related to cardiac dysfunction and ECM remodeling. Antioxidative stress therapy had beneficial effects by reducing diabetes‐reduced heart damage.^31^ Consistent with the above earlier evidences, our results showed a significant increase in T2D‐induced 4‐HNE expression levels and MDA, which were significantly reduced by the MH treatment. Therefore, our findings indicate that the MH treatment prevented diabetes‐induced cardiac inflammation and oxidative stress in T2D mice.

GSK‐3β is a serine/threonine kinase that is essential for the regulation of glycogen synthesis. T2D reduces GSK‐3β phosphorylation, which activates GSK‐3β, leading to inhibition of glycogen synthase, impaired glycogen synthesis and eventually insulin resistance.^32^ MH treatment increased the phosphorylation of GSK‐3β which was previously reduced by T2D. This may be the mechanism by which MH improves insulin resistance, which is consistent with our previous findings.^12^ The outer mitochondrial membrane enzyme CPT1 represents the initial and regulated step in the beta‐oxidation of FA, being the rate‐limiting enzyme for mitochondrial uptake of FA. Studies have shown that the up‐regulation of CPT1 caused by diabetes can enhance mitochondrial β oxidation in skeletal muscles, increase the utilization of FA as an energy source, lead to a decrease in lipid metabolite accumulation, and thus reduce the lipotoxicity in skeletal muscles.^33^ In this study, we found that T2D group of mice had lower expression of CPT1 protein than the Ctrl group. The MH treatment inhibited this decrease in CPT1.

T2D was earlier established to activate PPARα, a transcription factor that up‐regulates the uptake and metabolism of myocardial free fatty acids, leading to excessive accumulation of FA in cardiomyocytes.^34^ The treatment with MH in this study improved these unfavourable changes, including elevated CPT1 and decreased PPARα. AMPKα regulates cellular lipid and glucose metabolism. It controls the FA metabolism by activating PPARα.^35^ Our results indicate that MH can activate AMPKα in T2D mice. In addition, the activated AMPK further activates SIRT1, which is one of the key markers for the treatment of diabetes‐related fatty liver and alcoholic fatty liver disease.^36^, ^37^ Our experimental results also showed that MH significantly inhibited the decline of sirt1 in T2D mice. We found that MH could preserve normal Sirt1 level and stimulate AMPK levels in diabetic condition, which was in accordance with a recent study, where they used MH in cell cultured model and showing a similar result.^38^ We confirmed that the MH treatment regulated lipid accumulation by activating the AMPK/SIRT1/PPARα pathway, and protected against lipid accumulation leading to cardiac damage.

Taken together, our findings demonstrated the preventive effect of MH on DCM in T2D mice via the activation of AMPK/SIRT1/PPAR‐α pathway, regulation of lipid dysfunction, and attenuated cardiac oxidative stress, fibrosis, and inflammation in DCM (Figure 6). These findings may provide a potential opportunity for MH as a natural herbal medicine to prevent diabetic complications such as DCM for diabetic patients, which needs to be further explored in the future study. MH has bright prospects to be repurposed for future clinical use for the management of DCM and other cardiovascular complications of diabetes.

## CONFLICTS OF INTEREST

The authors declare that they have no conflicts of interest.

## AUTHORS’ CONTRIBUTIONS

ZYZ, LQB, ZGZ and LC originally designed the project. TJM and ZYZ performed the experiments, analysed the data, and wrote the manuscript draft. XL and HG partially conducted experiments and data collection. LC was responsible for the scientific review and manuscript editing. LQB and ZGZ monitored the project progression and modified the experimental designs and manuscript revision. All authors approved the final version of the manuscript.
